# Self-rated mental health and race/ethnicity in the United States: support for the epidemiological paradox

**DOI:** 10.7717/peerj.2508

**Published:** 2016-09-22

**Authors:** Alexis R. Santos-Lozada

**Affiliations:** Department of Sociology and Criminology, Pennsylvania State University, State College, PA, United States

**Keywords:** Self-rated mental health, United States, Epidemiological paradox, Race/ethnicity

## Abstract

This paper evaluates racial/ethnic differences in self-rated mental health for adults in the United States, while controlling for demographic and socioeconomic characteristics as well as length of stay in the country. Using data from the 2010 National Health Interview Survey Cancer Control Supplement (NHIS-CCS), binomial logistic regression models are fit to estimate the association between race/ethnicity and poor/fair self-reported mental health among US Adults. The size of the analytical sample was 22,844 persons. Overall prevalence of poor/fair self-rated mental health was 7.72%, with lower prevalence among Hispanics (6.93%). Non-Hispanic blacks had the highest prevalence (10.38%). After controls for socioeconomic characteristics are incorporated in the models, Hispanics were found to have a lower probability of reporting poor/fair self-rated mental health in comparison to non-Hispanic whites (*OR* = 0.70; 95% CI [0.55–0.90]). No difference was found for other minority groups when compared to the reference group in the final model. Contrary to global self-rated health, Hispanics were found to have a lower probability of reporting poor/fair self-rated mental health in comparison to non-Hispanic whites. No difference was found for non-Hispanic blacks when they were compared to non-Hispanic whites. Self-rated mental health is therefore one case of a self-rating of health in which evidence supporting the epidemiological paradox is found among adults in the United States.

## Introduction

Self-rated health (SRH) is among the most frequently assessed health perceptions in epidemiological research (Eriksson, Undén & Elofsson, 2001) and to this day remains a common outcome in research on socioeconomic inequalities in health (Dowd & Zajacova, 2007). In the United States (US), SRH is commonly evaluated using a five-point scale on which individuals are asked to rate their health status (Jylha, 2009; Zajacova & Dowd, 2011). Research on health and ethnicity has brought with it extensive investigations dealing with the epidemiological paradox, or Hispanic paradox (Ruiz, Steffen & Smith, 2013). This paradox surfaces because Hispanics, despite a lower socioeconomic position and less access to care, exhibit lower mortality rates than non-Hispanic whites (Markides & Coreil, 1986; Markides & Eschbach, 2005) as well as lower incidence of chronic conditions (Huh, Prause & Dooley, 2008) and hypertension (Borrell, 2009). SRH has become of interest to researchers given that, despite the Hispanic paradox, it is one of the few outcomes in which Hispanics do not exhibit better or similar indicators than non-Hispanic whites. Even when issues supporting or arguing against the validity of SRH have been well documented (Lundberg & Manderbacka, 1996; Chandola & Jenkinson, 2000; Finch, 2002; Quesnel-Vallée, 2007; Schnittker & Bacak, 2014) and the associations of SRH with certain outcomes remain unclear from a conceptual perspective (Jylha, 2009), SRH remains a powerful predictor of subsequent mortality (Idler & Benyamini, 1997; McGee et al., 1999; Finch, 2002; Dowd & Zajacova, 2007; Schnittker & Bacak, 2014) and has also been associated with the prevalence of numerous health conditions (Manor, Matthews & Power, 2001; Wu et al., 2013).

## Racial/Ethnic Disparities and Mental Health

Despite the vast literature related to racial/ethnic disparities in SRH, little attention has been given to self-rated mental health (SRMH) and its relation with race and ethnicity (Kim et al., 2011). The availability of a SRMH measurement provides the opportunity to evaluate whether support exists for the Hispanic paradox in a self-rated item other than overall health (SRH).

Previous research regarding mental illness has found supportive evidence for the immigrant advantage with regard to US born individuals (Alegria et al., 2008). However, significant variation was observed within the Hispanic population. In the case of SRMH in the US, various studies have explored racial/ethnic differences in different contexts. Of these, two were based on small samples from specific residential settings or age groups. The first was the Collaborative Psychiatric Epidemiology Survey 2001–2003, in which only persons over 60 years old were included (Kim et al., 2011). In this survey, Hispanics were found to have a higher proportion of individuals reporting poor/fair SRMH than non-Hispanic whites. The second was a study conducted in Amarillo, Texas in which Hispanics were less likely to report good/very good/good SRMH when compared to non-Hispanic blacks (Rohrer, Pierce & Blackburn, 2005). In the only instance in which a nationally representative sample was analyzed, Hispanics were less likely to report poor/fair SRMH when compared to non-Hispanic whites. This association is not clear, as the model in this study also included an interaction between race/ethnicity and a mental component summary score (Zuvekas & Fleishman, 2008).

Individuals may take in consideration different factors in the process of rating their health. Cultural backgrounds, may impact the influence of socioeconomic status, household and community level characteristics for individuals when ratings of health (Bzostek, Goldman & Pebley, 2007). In addition, differences in the process of somatization could also play a role in mental health ratings. Through the process of somatization family support (familismo) has been found to be protective with regards to depression (Ayón, Marsiglia & Bermudez-Parsai, 2010). Culturally speaking mental health symptoms are found to be considered differently by the Hispanic community (Guarnaccia, Martinez & Acosta, 2005). In order to avoid the social stigma of being considered “loco” (crazy), mental health symptoms are labeled as “ataque de nervios” or just “nervios” (Liebowitz et al., 1994). Labeling mental health as a transient event avoids the negative connotation of a mental health diagnosis and being considered dangerous, violent and suffering from an incurable condition (Guarnaccia, Martinez & Acosta, 2005). Considering mental health conditions to be transient or fleeting may reduce the odds of rating mental health as poor for the Hispanic population.

Racial/ethnic disparities have been observed for non-Hispanic blacks, and the disadvantage that this group has in comparison to non-Hispanic whites. The disadvantage is attributed to differences in socioeconomic characteristics (Williams et al., 1997), residential segregation (Massey, 2004), and inhospitable and stressful environments (Jackson, Knight & Rafferty, 2010). Despite similar incidence rates of chronic major depressive disorder (MDD) non-Hispanic blacks were found to have higher risk of having chronic, very chronic and disabling levels paired with lower therapeutic interventions to treat their depression (Williams et al., 2007). Supportive evidence for similar or even lower risk of anxiety and panic disorders as well as social anxiety were found when comparing non-Hispanic whites with Caribbean blacks and non-Hispanic blacks (Himle et al., 2009). Similar to the study regarding MDD, non-Hispanic blacks and Caribbean blacks were found to have higher degree of severity related to anxiety and panic, as well as in functional limitations when they were compared to non-Hispanic whites (Himle et al., 2009).

This paper has two aims: (1) to assess whether the epidemiological paradox extends to SRMH or whether, as in the case of SRH, the paradox is not present and (2) to study differences in ratings between non-Hispanic blacks and non-Hispanic whites. I hypothesize that the odds of poor/fair SRMH will be lower for Hispanics when compared to non-Hispanic whites, especially when controlling for demographic, socioeconomic and acculturation measures (Hypothesis 1). This hypothesis is proposed for two main reasons: the concept of “familismo” or family support seems to act as a support mechanism for the process of somatization for members of the Hispanic community; in addition, the notion of mental illness or conditions being temporary or transient also supports the expectation of best SRMH. Those two circumstances would operate towards making Hispanics less likely to report poor/fair SRMH, in accordance to the epidemiological paradox. Additionally, I hypothesize that non-Hispanic blacks will have higher odds of reporting poor/fair SRMH when compared to non-Hispanic whites, even when controlling for demographic, socioeconomic, and acculturation measures (Hypothesis 2). Given the diversity of the non-Hispanic others group (Asian, Native-American, Pacific Islanders, mixed backgrounds, etc.); no hypothesis is postulated for this group. Below, I detail the data and statistical methods used to test these hypotheses.

## Methods

### Data and measures

Data for this study come from the National Health Interview Survey (NHIS). The main objective of the NHIS is to monitor the health of the civilian noninstitutionalized population of the United States through the collection and analysis of data on a broad range of health issues (Schiller et al., 2012). I use the 2010 Cancer Control Supplement (NHIS-CCS), which collects information from a random sub-sample of adults who participate in the NHIS on issues pertaining to knowledge, attitudes, and practices in cancer-related health behaviors (National Cancer Institute, 2015). Those who are administered the supplement also answer questions about self-rated health in both the general (SRH) and mental aspects (SRMH). Supplemental weights are computed to allow the results of the analysis performed on the supplement data to be generalizable to the non-institutionalized adult US population.

A total of 27,157 individuals were administered the supplement. I employed listwise deletion for both the dependent and independent variables for instances in which missing values were found. The size of the final analytical sample consisted of 22,844 individuals with valid information for the variables considered in the study, which represents a reduction of 4,313, or 15.88%, of the initial sample. 97.24% of the deleted observations were dropped because of missing values on one of two variables. The biggest source of this drop in the sample size came from the source variable for the outcome, which accounted for 2,253 observations, or 52.24% of the cases that were eliminated. The second variable that caused the elimination of a high percentage of the observations was the position of the individual in relation to the poverty threshold, which accounted for 1,941 cases, or 45.00% of the cases that were eliminated. This is common, as income level is one of the variables where respondents are less likely to answer (Kim et al., 2007). Sensitivity tests were performed through the imputation of this variable avoiding the elimination of these cases; the results from the empirical models were not altered by the inclusion/exclusion of these observations. The remaining 2.76% of the cases that were eliminated were accounted for by: education (57 observations or 1.32%), time in the United States (35 observations or 0.81%), and marital status (27 observations or 0.63%).

### Self-rated mental health

The key variable of this analysis was SRMH. This variable was collected by asking the respondents to rate their general mental health, including their mood and ability to think. The respondents could select one of five categories: ‘Excellent,’ ‘Very good,’ ‘Good,’ ‘Fair,’ and ‘Poor.’ I followed the usual dichotomization that is used through most SRH literature, wherein zero represents those who rate their health as Excellent, Very Good, and Good, and one indicates that the respondents rated their health as poor or fair (Idler & Benyamini, 1997; Subramanian, Acevedo-Garcia & Osypuk, 2005; Acevedo-Garcia et al., 2010). Individuals who refused or expressed not knowing how to answer the question were excluded from the analysis; the magnitude of the exclusion was discussed in the previous section.

### Demographic and socioeconomic characteristics and assimilation

The demographic and socioeconomic characteristics incorporated in this analysis were: race/ethnicity, age, sex, educational attainment, position with regards to the poverty threshold, and marital status. Race/ethnicity was measured as a categorical variable indicating whether the respondent was: non-Hispanic white, non-Hispanic black, Hispanic, or non-Hispanic other. Three age categories were generated from a continuous source variable; the new variable included the categories 18–44 years, 45–64 years, and 65 years of age or older. Sex was a dichotomous variable indicating whether the respondent was male or female. Educational attainment was incorporated as a four-category variable that indicated whether the respondent had not completed high school (including 12th grade but without graduation), only completed high school or earned a GED, completed an associate’s degree or some years of college, or completed a college degree or more. Poverty status was measured as a dichotomous variable that indicated whether the respondent lived below or at/above the poverty threshold. Marital status was measured as a five-category variable indicating whether at the moment of the survey the respondent was: Single/Never Married, Married, Widowed, Divorced, or Separated. Time spent in the US was incorporated to account for assimilation into US culture. This variable was measured in three categories, indicating whether the respondent was born in the US, had spent 0–14 years in the US, or had spent more than 14 years in the country. The measurement of time in the United States is included given the role it has been found to have in SRH and the patterns of health deterioration found in previous published work (Acevedo-Garcia et al., 2010; Hamilton & Hummer, 2011). Previous research has also recommended controlling for acculturation when using SRH, especially in the case of Hispanics (Finch, 2002).

## Statistics

Weight variables were included with the NHIS-CCS data to account for the complex sample design and adjust for probabilities of the selections of respondents, disproportionate selection of subgroups, and non-response. The survey procedures in SAS 9.4 (SAS Institute, Cary, NC, USA) were used to incorporate these design effects into the calculation of point estimations and standard errors in both the descriptive and inf erential statistics. Due to the complex sample design of the NHIS-CCS, I used the SURVEYFREQ procedure in SAS 9.4 to adjust for survey design when calculating the descriptive statistics and the Rao-Scott Chi-Square for the tests of association.

Logistic regression models with adjustments for survey design were estimated to establish the association between race/ethnicity and poor/fair SRMH and each of the covariates detailed previously. These models were estimated in a nested manner in order to observe differences in the association between race/ethnicity and the outcome and then to observe differences with each set of additional variables. These models were estimated using the SURVEYLOGISTIC procedure in SAS 9.4 in order to adjust the standard errors and the subsequent estimates of significance. The results are presented as odds ratios, confidence intervals, and levels of significance for all variables included in the models. The extent to which the addition of variables improved model fit was assessed through the Akaike Information Criteria (AIC). The AIC reduced with the addition of each set of covariates, meaning each model explains the outcome better than the previous one. Additionally, I explored the possibility of multicollinearity among the variables included in the models using the REG procedure in SAS 9.4, adjusting for survey weights. The values of the variance inflation factor (VIF) for each independent variable indicated that multicollinearity was not present in the multivariable models.

## Results

### Descriptive statistics

Table 1 presents the distribution and test of significance for the outcome variable and all covariates, as well as Chi-Square tests of association by race/ethnicity. Overall, 7.72% of the population reported poor/fair SRMH. Significant differences were found for poor/fair SRMH by race/ethnicity (*p* ≤ 0.0001). Non-Hispanic whites reported the outcome in a proportion of 7.64%, while non-Hispanic blacks reported it in a higher proportion of 10.38%. Both Hispanics and non-Hispanic others reported poor/fair SRMH at a lower proportion than non-Hispanic whites (6.93% and 4.98%, respectively).

**Table 1:** Weighted descriptive statistics for Poor/Fair Self-Rated Mental Health, demographic and socioeconomic characteristics, length of stay in the country and by race/ethnicity for Adults in the United States, National Health Interview Survey-Cancer Control Supplement 2010 (*n* = 22, 844).

	Overall sample	Non-hispanic whites	Non-hispanic blacks	Hispanics	Non-hispanic others	Chi-square	*p*-value
**Self-rated mental health**							
Poor/fair SRMH	7.72	7.64	10.38	6.93	4.98	40.95	<0.0001
**Race/ethnicity**							
Non-Hispanic white	68.98	–	–	–	–	–	–
Non-Hispanic black	11.59	–	–	–	–		
Hispanic	13.92	–	–	–	–		
Non-Hispanic other	5.52	–	–	–	–		
**Age**							
18–44 years	49.80	44.97	56.69	65.30	56.58	484.04	<0.0001
45–64 years	34.78	37.06	32.23	26.74	31.86		
65 years of age or older	15.42	17.97	11.10	7.96	11.56		
**Sex**							
Male	48.83	49.08	44.98	51.96	46.00	28.02	<0.0001
Female	51.17	50.92	55.02	48.04	54.00		
**Education**							
Less than high school	13.94	9.19	17.32	36.92	8.26	1,707.39	<0.0001
High school diploma	25.92	25.88	29.89	25.23	19.66		
Associate degree or some college	30.94	32.16	34.67	24.29	24.67		
College or more	29.21	32.77	18.12	13.56	47.42		
**Poverty threshold**							
Below poverty threshold	13.25	9.03	24.83	23.97	14.67	645.80	<0.0001
At or above poverty threshold	86.75	90.97	78.17	76.03	85.33		
**Marital status**							
Single/never married	26.09	22.67	43.09	29.35	24.83	840.59	<0.0001
Married	55.08	57.80	34.65	55.10	63.94		
Widowed	5.44	5.94	5.73	3.31	3.94		
Divorced	10.91	11.72	12.20	7.86	5.82		
Separated	2.48	1.87	4.33	4.38	1.48		
**Years in the United States**							
US born	83.15	94.68	89.00	40.35	34.61	4,891.76	<0.0001
0–14 years	6.37	1.71	4.70	23.16	25.74		
Over 14 years	10.48	3.60	6.30	36.49	39.65		
***Unweighted n***	22,844	13,202	3,683	4,323	1,636		

**Notes.**

**Survey Design:** PSU = PSU, STRATA = STRATA, WEIGHT = SAMPWEIGHT.

Significant differences were also found for the distribution of all the variables included in the analysis by race/ethnicity. Hispanics had a higher proportion of persons in the youngest age group (65.30%). Male/Female ratios seem to gravitate around 50-50 for all groups, except for non-Hispanic blacks and non-Hispanic others where the sample was female dominant (55% and 54%, respectively). A higher proportion of non-Hispanic whites (32.77%) and non-Hispanic Others (47.42%) had completed a college degree or more. In the case of non-Hispanic blacks most of the participants had an associate degree or some college studies (34.67%) while most of the Hispanic respondents had not completed a high school diploma (36.92%). Poverty rates are higher for non-Hispanic blacks (24.83%) and Hispanics (23.97%), in comparison to the poverty rates for non-Hispanic whites and non-Hispanic others (9.03% and 14.67%, respectively). Differences in marital status are found for each race/ethnic group, with non-Hispanic whites, Hispanics and non-Hispanic others having higher proportions of the population in the married group (57.80%, 55.10%, and 63.94%, respectively). A higher proportion of non-Hispanic blacks were found to be Single/Never Married (43.09%). Finally, lower proportions of US born population are found for Hispanics and non-Hispanic others (40.35% and 34.61%, respectively) when compared to approximately 90% for the other two race/ethnic groups.

### Regression models

Results from the logistic regression models are presented in Table 2. When race/ethnicity was included in Model 1, non-Hispanic blacks were found to be at 40% higher odds of reporting poor/fair SRMH compared to non-Hispanic whites. In turn, non-Hispanic others were found to be at 37% lower odds of reporting the same outcome when compared to non-Hispanic whites. No difference was found for Hispanics when compared to the reference group on this univariate model.

**Table 2:** Weighted regression models for Odds of Poor/Fair Self-Rated Mental Health race/ethnicity, demographic and socioeconomic characteristics, and length of stay in the country for Adults in the United States, National Health Interview Survey-Cancer Control Supplement 2010 (*n* = 22, 844).

	Model 1		Model 2		Model 3		Model 4	
	OR (95 % CI)		OR (95% CI)		OR (95% CI)		OR (95% CI)	
**Race/Ethnicity**								
Non-Hispanic white	1.00	–	1.00	–	1.00	–	1.00	–
Non-Hispanic black	1.40 (1.19–1.64)	^***^	1.27 (1.08–1.50)	^**^	0.95 (0.80–1.13)		0.97 (0.81–1.15)	
Hispanic	0.90 (0.77–1.05)		0.97 (0.83–1.13)		0.57 (0.47–0.68)	^***^	0.70 (0.55–0.90)	^**^
Non-Hispanic other	0.63 (0.50–0.80)	^***^	0.69 (0.54–0.88)	^**^	0.68 (0.53–0.87)	^**^	0.83 (0.62–1.10)	
**Age**								
18–44 years			1.00	–	1.00	–	1.00	–
45–64 years			1.70 (1.47–1.96)	^***^	1.74 (1.50–2.01)	^***^	1.68 (1.45–1.95)	^***^
65 years of age or older			1.32 (1.11–1.58)	^**^	1.17 (0.97–1.40)	^a^	1.12 (0.93–1.35)	
**Sex**								
Male			1.00	–	1.00	–	1.00	–
Female			1.13 (1.00–1.27)	^*^	1.13 (1.00–1.27)	^*^	1.13 (1.00–1.27)	^*^
**Education**								
Less than high school					1.00	–	1.00	–
High school diploma					0.55 (0.47–0.64)	^***^	0.54 (0.46–0.63)	^***^
Associate degree or some college					0.39 (0.33–0.46)	^***^	0.38 (0.32–0.45)	^***^
College or more					0.23 (0.19–0.29)	^***^	0.23 (0.19–0.28)	^***^
**Poverty threshold**								
Below poverty threshold					2.24 (1.95–2.58)	^***^	2.30 (2.00–2.64)	^***^
At or above poverty threshold					1.00	–	1.00	–
**Marital status**								
Single/never married			1.00	–	1.00	–	1.00	–
Married			0.55 (0.48–0.64)	^***^	0.68 (0.58–0.80)	^***^	0.71 (0.60–0.83)	^***^
Widowed			1.11 (0.87–1.42)		1.02 (0.77–1.34)		1.06 (0.82–1.36)	
Divorced			1.15 (0.96–1.38)		1.23 (1.02–1.48)	^**^	1.25 (1.04–1.51)	^**^
Separated			1.20 (0.92–1.55)		1.01 (0.77–1.34)		1.04 (0.79–1.37)	
**Years in the United States**								
US born							1.00	–
0–14 years							0.45 (0.32–0.62)	^***^
Over 14 years							0.82 (0.62–1.09)	
**Intercept**	−1.91	^***^	−2.55	^***^	−1.89	^***^	−1.89	^***^
**AIC**	104,846,694		102,762,924		97,776,180		97,508,330	

**Notes.**

**Survey Design:** PSU = PSU, STRATA = STRATA, WEIGHT = SAMPWEIGHT.

a ≤0.10.

* *P* ≤ 0.05.

** *P* ≤ 0.01.

*** *P* ≤ 0.001.

Age, sex, and marital status were added in Model 2. The racial/ethnic differences weakened, but remained significant with the inclusion of these variables. In Model 2, non-Hispanic blacks were found to be at 27% higher odds of reporting poor/fair SRMH when compared to non-Hispanic whites. No difference was found for odds of reporting poor/fair SRMH for Hispanics when compared to non-Hispanic whites. Non-Hispanic others continued to have lower odds of reporting poor/fair SRMH when compared to the reference group. Individuals in the middle age group (45–64) were at higher odds (OR = 1.70) of reporting poor/fair SRMH, while those aged 65 and older had significant higher odds of reporting the outcome in relation to those aged 18–44 years (OR = 1.32, *p* = 0.002). Females were at higher odds of reporting poor/fair SRMH when compared to males, equivalent to a 13% higher probability. For marital status, married individuals were found to be at 45% lower odds of reporting poor/fair SRMH when compared to individuals who have never been married (Single). No difference was found for the other marital statuses

Education and position with regards to the poverty threshold were added in Model 3. The differences for non-Hispanic blacks weakened to a non-significant level. Hispanics were found to be at lower odds of reporting poor/fair SRMH when compared to non-Hispanic whites (OR = 0.57, *p* < 0.0001). The differences for non-Hispanic others remained strong in Model 3. The associations between age, sex, and marital status are comparable to those found in Model 2. A significant and increasingly protective effect was observed for higher levels of educational attainment, with individuals in groups with higher education having a lower probability of reporting poor/fair SRMH when compared to those with less than a high school education. Also, individuals who lived below the poverty threshold were at significantly higher odds of reporting the outcome in contrast to those who lived above it (OR = 2.24). Finally, those who were married were at lower odds of reporting the outcome when compared to those who were single/never married (OR = 0.68). Being divorced was significantly associated with higher probability of reporting poor/fair SRMH (OR = 1.23). No difference was observed for those who were widowed or separated when compared to those who were single/never married.

Time in the US was incorporated in Model 4, and the association between race/ethnicity and reporting poor/fair SRMH weakened in this model. As in Model 3, no difference was observed for non-Hispanic blacks when compared to the reference group. Hispanics had 30% lower odds of reporting poor/fair SRMH when compared to non-Hispanic whites. This was the only race/ethnic group that remained statistically different when compared to the reference group after the inclusion of demographic, socioeconomic and acculturation controls. The difference between non-Hispanic others and non-Hispanic whites disappeared with the addition to the model of years in the US. No changes in the associations between the outcome and age, sex, education, poverty, or marital status were observed after the factor of years in the US was incorporated into Model 4. Individuals who had lived in the US between 0–14 years had 55% lower odds of reporting poor/fair SRMH when compared to those born in the US. No difference was observed for persons who had lived in the US for more than 14 years in comparison to those born in the US.

## Discussion and Conclusions

This paper fills a clear gap in research regarding self-rated health and race/ethnicity by extending the knowledge in the area of SRMH. Multiple results from this paper are worthy of discussion and further analysis, especially the discovery that racial/ethnic groups rate their mental health at different levels and in a significantly different way. In relation to the hypotheses that guided this study, support was found for the first hypothesis, with Hispanics having lower odds of reporting poor/fair SRMH when compared to non-Hispanic whites. This finding is highly relevant given that Hispanics have been found to rate their health (general or global health) at worse levels than non-Hispanic whites (Cho et al., 2004; Bzostek, Goldman & Pebley, 2007; Kandula, Lauderdale & Baker, 2007; Viruell-Fuentes et al., 2011). The results provide support for the epidemiological paradox and represents one case in which Hispanics exhibit similar (Models 1 and 2) or lower odds (Models 3 and 4) in a poor/fair rating of their health in comparison to non-Hispanic whites. It is important to highlight that the instances where Hispanics have lower odds of reporting poor/fair SRMH are those where controls for socioeconomic characteristics are incorporated (Models 3 and 4). In relation to the second hypothesis, which postulated that non-Hispanic blacks would have higher odds of reporting poor/fair SRMH, initial support was found for this hypothesis (Model 1 and Model 2); however, the difference disappears after controlling for socioeconomic characteristics (Models 3 and 4). These results are puzzling, as non-Hispanic blacks regularly exhibit more severe mental health outcomes than non-Hispanic whites, and this extends to the usual SRH measures but does not seem to extend to SRMH. The absence of differences for this particular outcome should be explored in future studies. Previous research has found differences in the prevalence of dysthymic disorder with race/ethnicity when comparing non-Hispanic blacks with non-Hispanic whites (Riolo et al., 2005). Minority groups were found to have higher rates of dysthymic disorder, but once socioeconomic and demographic characteristics are controlled the odds of having this disorder reverse. Among these, education and poverty status are highlighted as important intervening variables. In addition to dysthymic disorder, the study also finds differences in prevalence of major depressive disorders, with minority groups having lower prevalence. However, similarity in the incidence of other mental health conditions such as anxiety disorders and major depressive disorders have been documented previously (Williams et al., 2007; Himle et al., 2009) The results of this study provide additional support for the no black-white difference with regards to a mental health outcome once demographic and socioeconomic characteristics are accounted for in the empirical models (Models 3 and 4).

No specific hypothesis was postulated for non-Hispanic others, given the diversity of individuals included in this race/ethnic group. In Models 1, 2 and 3, non-Hispanic others had lower odds of reporting poor/fair SRMH (37%, 31%, and 32% lower probability, respectively) when compared to non-Hispanic whites. In Model 4, this association weakened to a non-significant level. No further conclusions can be reached with regards to this group, as it is composed of individuals from multiple racial backgrounds (Asian, Native-American, Pacific Islanders, mixed backgrounds, etc.).

What about the additional covariates? Some of the usual associations found for SRH also extend to SRMH. A clear example is women rating their health worse than men; this is consistent with documented patterns in health literature (Case & Paxson, 2005; Liu & Hummer, 2008). The association between higher educational attainment and decreasing odds of reporting poor/fair SRMH is also consistent with the protective effect of educations with regards to SRH (Mirowsky & Ross, 2008; Hu & Hibel, 2013). The hazardous nature of living below the poverty threshold also extends to SRMH, which is consistent with the effect of this variable found throughout the health disparities and SRH literature (Kawachi, Kennedy & Glass, 1999). In relation to marital status, the results are found to be consistent with previous research on health outcomes, SRH, and even mortality. Marriage acts as a protective element against poor/fair SRMH, as it does for SRH, risky behaviors, and mortality risk (Rendall et al., 2011). Furthermore, the detrimental effect of marital disruption is also found for poor/fair SRMH, as has been documented for SRH (Lindstrom, 2009) and mortality risk (Manzoli et al., 2007). Divorced individuals were at higher odds of reporting poor/fair SRMH, and those who are widowed or separated are found to be no different in reporting poor/fair SRMH than those who are single/never married.

An even more interesting result is the pattern observed in Model 4 in relation to years in the United States. When immigrants who have spent between 1 and 14 years in the US are compared to US-born individuals, they are at a lower probability of reporting poor/fair SRMH. On the other hand, immigrants in the group that has spent more than 14 years in the US are no different than those born in the US with regards to poor/fair SRMH. I found support for health deterioration as length of residence in the United States increases. Numerous studies have found support for a trend in which individuals who arrive in the United States experience a deterioration in their health as they spend more time in the country (Frisbie, Cho & Hummer, 2001; Finch, 2002; Cho et al., 2004; Hamilton & Hummer, 2011).

This analysis is subject to various limitations. A control for language of interview was not included in the analysis. Given the issues with the validity of SRH comparisons in multilingual societies, this would have been justifiable based on previous evidence that examines language and SRH. A univariate regression model was used estimate the association between language of interview and poor/fair SRMH, none of the empirical tests were significant (results not shown), and the total number of respondents who answered in a language other than English represented less than 6% of the weighted population. Among them, some individuals took the survey in a language that was neither English nor Spanish, and their elimination would have caused a reduction on the size of the final analytical sample. Additionally, the results are based in cross-sectional data from which means causality cannot be inferred.

In conclusion, this paper presents a SRH item in which Hispanics exhibit favorable outcomes when compared with non-Hispanic whites. The implications are multiple, as it seems that the absence of support for the epidemiological paradox in self-ratings of health does not extend through all the self-ratings of health. Future research could study the underlying causes of this finding as well as how the diagnosis of health conditions affects these ratings. Ultimately, the limitations of this study and the potential of future research both go back to the limited instances in which data about SRMH are collected. Performing a comparative study with a survey that asks about both SRH and SRMH and studying how demographic, socioeconomic, acculturation, and health behaviors and conditions affect each outcome could shed light as to why Hispanics rate their health (SRH) worse than non-Hispanic whites but not their mental health (SRMH).

## Supplemental Information

10.7717/peerj.2508/supp-1 Supplemental Information 1 Dataset for Analysis

10.7717/peerj.2508/supp-2 Supplemental Information 2 Codebook for the dataset Codebook for PeerJ_dotMental.csv and used for the analysis included in the paper.

10.7717/peerj.2508/supp-3 Supplemental Information 3 Code for Empirical Models

## Additional Information and Declarations

### Data Availability

The following information was supplied regarding data availability: The raw data (the data code, a codebook) has been supplied as Supplementary Files.
