# Supplementary material for: Self-rated mental health and race/ethnicity in the United States: support for the epidemiological paradox
Source: PeerJ. 2016 Sep 22;4:e2508. doi: 10.7717/peerj.2508 (PMC5036069; doi:10.7717/peerj.2508)
Supplement: Supplemental Information 2 — Codebook for PeerJ_dotMental.csv and used for the analysis included in the paper. [file peerj-04-2508-s002.docx]

**CODEBOOK FOR SELF-RATED MENTAL HEALTH AND RACE/ETHNICITY IN THE UNITED STATES: SUPPORT FOR THE EPIDEMIOLOGICAL PARADOX**

| **Variable**  (Variable Name) | **Codebooks** | **Reference Group**  Omitted in the regression model |
| --- | --- | --- |
| Self-Rated Mental Health  POORSRHMENT | 1=POOR  0=GOOD | GOOD (0) |
| RACE  (RACE) | WHITE  BLACK  HISPANIC  OTHER | WHITE |
| Poverty Threshold  (POOR) | 0 = At or above the poverty threshold  1= Below the poverty threshold | At or above the poverty threshold (0) |
| Sex  (FEMALE) | 0=Male  1=Female | Male |
| Marital Status  (MARGRP) | 1=Married (MARRIED)  5=Widowed (WIDOWED)  3=Divorced (DIVORCED)  4=Separated (SEPARARED)  1=Never Married/Single | Single |
| Education (EDUCA) | Less HS  High School (HS)  AA Degree/Some col  College or More | Less HS |
| AGE (AGGRP) | 1 = 18 - 44  2 = 45- 64  3 = 65 + | 18 - 44 |
| Education (EDUCA) | Less HS  HS (High School)  AA Degree/Some college  College or more | Less HS |
| Years in the United States  (MIGGRP) | 1 = U.S. Born  2 = 1 - 15 years  3 = Over 15 years | U.S. Born |
